# Acute toxicity and genotoxicity assessment of environmental contaminants using *Artemia salina*

**DOI:** 10.2478/aiht-2026-77-4111

**Published:** 2026-06-30

**Authors:** Jelena Đorđević Aleksić, Luka Gačić, Margareta Kračun-Kolarević, Jovana Jovanović Marić, Branka Vuković-Gačić, Stoimir Kolarević

**Affiliations:** University of Belgrade, Institute for Multidisciplinary Research – National Institute of the Republic of Serbia, Belgrade, Serbia; University of Belgrade Faculty of Agriculture, Belgrade, Serbia; University of Belgrade, Institute for Biological Research “Siniša Stanković” – National Institute of the Republic of Serbia, Belgrade, Serbia; University of Belgrade Faculty of Biology, Centre for Genotoxicology and Ecogenotoxicology, Belgrade, Serbia

**Keywords:** 5-fluorouracil, benzalkonium chloride, brine shrimp, cisplatin, comet assay, cytotoxicity, etoposide, glyphosate, LC_50_, tributyltin chloride, 5-fluorouracil, benzalkonijev klorid, cisplatin, citotoksičnost, etopozid, glifosat, komet-test, LC_50_, tributilkositrov klorid

## Abstract

The widespread presence of pharmaceuticals and industrial pollutants in aquatic environments necessitates rapid screening tools capable of detecting hazards and adverse biological effects. In this study, we selected the nauplii of *Artemia salina* (brine shrimp) as a model to screen for the acute toxicity and genotoxicity of three cytostatics (5-fluorouracil, cisplatin, and etoposide), one pesticide (glyphosate), one surfactant (benzalkonium chloride), and one antifouling agent (tributyltin chloride), all applied in graded concentrations for 24 and 48 h. Acute toxicity was quantified by determining median lethal concentrations (LC_50_), and the genotoxicity of cytostatics was assessed using the alkaline comet assay. All substances caused concentration and time-dependent mortality. Among cytostatics, cisplatin was the most toxic (48 h LC_50_≈35 µg/mL), followed by etoposide (~130 µg/mL), while 5-fluorouracil was the least toxic (>500 µg/mL). Tributyltin chloride showed the strongest effect among non-pharmaceuticals (0.55 µg/mL), followed by benzalkonium chloride (~7.5 µg/mL) and glyphosate (~43 µg/mL). Cytostatics induced concentration-dependent DNA damage, with etoposide exhibiting the highest tail intensity (~60 %). These findings demonstrate that *A. salina* is an effective model for early, cost-effective hazard screening and highlight the importance of including genotoxic endpoints, particularly when assessing chemicals with DNA-targeting mechanisms.

## Abstract (hr)

Sveprisutnost farmaceutika i industrijskih onečišćivala u vodenim okolišima zahtijeva brze alate za probir koji mogu otkriti opasnosti i štetne biološke učinke. U ovom istraživanju odabrani su naupliji vrste *Artemia salina* (slanišni škrgonožac) kao model za ispitivanje akutne toksičnosti i genotoksičnosti triju citostatika (5-fluorouracil, cisplatin i etopozid), jednog pesticida (glifosat), jednog surfaktanta (benzalkonijev klorid) i jednog sredstva protiv obraštanja (tributilkositrov klorid), pri čemu su sve tvari primijenjene u gradiranim koncentracijama tijekom 24 i 48 sati. Akutna toksičnost kvantificirana je određivanjem srednjih letalnih koncentracija (LC_50_), a genotoksičnost citostatika procijenjena je primjenom alkalnoga komet-testa. Sve ispitivane tvari prouzročile su smrtnost ovisnu o koncentraciji i vremenu izloženosti. Među citostaticima je najtoksičniji bio cisplatin (48 h LC_50_≈35 µg/mL), zatim etopozid (~130 µg/mL), a 5-fluorouracil bio je najmanje toksičan (>500 µg/mL). Među nefarmaceutskim tvarima je najizraženiji učinak pokazao tributilkositrov klorid (0,55 µg/mL), a slijedili su ga benzalkonijev klorid (~7,5 µg/mL) i glifosat (~43 µg/mL). Citostatici su prouzročili oštećenje DNA ovisno o koncentraciji, pri čemu je etopozid pokazao najveći intenzitet repa (>60 %). Ovi rezultati potvrđuju da je vrsta *A. salina* pogodna za rani i učinkovit probir opasnosti te naglašavaju važnost uključivanja pokazatelja genotoksičnosti, osobito pri procjeni kemikalija čiji mehanizmi djelovanja ciljaju na DNA.

In recent years, increasing attention has been given to environmental contamination by a wide range of chemical substances, including pharmaceuticals, pesticides, and industrial pollutants (1,2,3,4). Every year, hundreds of new chemical compounds are introduced into global production and use, many of which ultimately reach aquatic ecosystems (5, 6). Furthermore, many cytostatics and pesticides are now more potent and more resistant to degradation, exerting toxic and/or genotoxic effects on non-target organisms at very low concentrations due to selective action on specific proteins (7, 8).

In this context, there is a growing need for rapid and cost-effective screening tools capable of early detection of potential hazards and adverse biological effects. Over the past decades, various invertebrate species have been widely used in ecotoxicological studies due to their sensitivity to numerous physical and chemical agents. Among them, the brine shrimp *Artemia* spp. have emerged as a suitable and frequently applied model organism for toxicity testing due to their short life cycle, cost-effectiveness, short exposure time (24–72 h), and flexibility of application across diverse chemical classes (9,10,11). They inhabit hypersaline environments worldwide, and their nauplii are often used as live feed in aquaculture and aquaria (12, 13). Being available as cysts, easy to cultivate, and producing homogeneous populations makes them reliable and convenient for laboratory bioassays, yet their potential for both general toxicity and early hazard screening, particularly for genotoxicity assessment, has remained insufficiently explored.

To address this gap, we decided to test the potential of brine shrimp as a model screening tool across different classes of chemicals (cytostatics, pesticides, surfactants, and antifouling agents) to get a broader idea of its responsiveness and set up a comparative framework for interpreting toxic effects across compounds with distinct modes of action. The central hypothesis was that *A. salina* could serve as a reliable, cost-effective model organism for rapid screening of both acute toxicity and genotoxicity and that its sensitivity would depend on the mode of action of the tested compounds.

## MATERIALS AND METHODS

For this purpose, we evaluated the acute toxicity of three cytostatics (5-fluorouracil, cisplatin, and etoposide), one pesticide (glyphosate), one surfactant (benzalkonium chloride), and one antifouling agent (tributyltin chloride) using *A. salina* larvae following the protocol of the Artemia Reference Centre (ARC) test developed at Ghent University, Belgium. While all six substances were tested for their general toxic effects, genotoxicity assessment was specifically conducted for the cytostatics, given their well-known capacity to interact directly with DNA and thus pose a greater risk to genomic integrity than pesticides and other environmental contaminants (14).

Acridine orange, dimethyl sulphoxide (DMSO) solution, normal melting point agarose (NMP), low melting point agarose (LMP), ethylenediaminetetraacetic acid (EDTA), sodium hydroxide, sodium chloride, Triton X-100, Tris, 5-fluorouracil (CAS No. 51-21-8), cisplatin (CAS No. 15663-27-1), etoposide (CAS No. 33419-42-0), benzalkonium chloride (CAS No. 63449-41-2), and tributyltin chloride (CAS No. 1461-22-9) were purchased from Sigma-Aldrich (St. Louis, MO, USA). Potassium dichromate (CAS No. 7778-50-9) was purchased from VWR International (Radnor, PA, USA). Glyphosate (Glifosav-480, with the active 360 g/L glyphosate-IPA salt as active substance) was purchased from Agrosava (Šimanovci, Serbia). Dehydrated *A. salina* eggs were purchased under the brand name Artemia Eggs from Dajana (Bohuňovice, Czechia, EAN: 8594000251330). Sea salt was purchased from Aquaforest (Brzesko, Poland).

### *A. salina* culture

Commercially available *A. salina* eggs (approximately 0.3 g) were placed in 500 mL of freshly prepared artificial seawater containing 33 g of sea salt per 1000 mL of distilled water. With intensive aeration and illumination with a 100 W (4000 K) lamp, the mixture was incubated at 25 °C for 24 h. After incubation, aeration was turned off, the hatched larvae (stages 1 and 2) were collected using phototaxis and transferred to freshly prepared artificial seawater. Viable larvae (stage 2) were extracted and relocated to 24-well plates (10 specimens per well containing 1 mL of seawater) for toxicity testing or 50 mL polystyrene beakers for genotoxicity testing.

### Artemia Reference Centre (ARC) test

**Figure 1:**
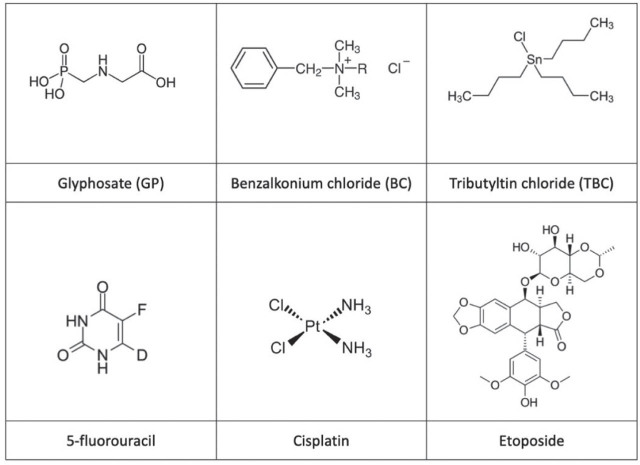
Chemical structure of the tested compounds

Figure 1 shows the chemical structure of the compounds used in this study. Stock solutions of the tested cytostatics were prepared prior to the experiment. Glyphosate, benzalkonium chloride, potassium dichromate, 5-fluorouracil, and cisplatin were directly dissolved in seawater due to good water solubility. Tributyltin chloride and etoposide were first dissolved in DMSO and diluted with seawater to obtain the desired test concentrations. The final concentration of DMSO in all treatments did not exceed 0.1 %.

Test solutions were added to the wells and the plates incubated in the dark at 25 °C for 4 h, 24 h, and 48 h. For each compound, six concentrations in a two-fold dilution series were tested. The highest tested concentrations were 1000 µg/mL for 5-fluorouracil, 50 µg/mL for cisplatin, 500 µg/mL for etoposide, 300 µg/mL for glyphosate, 31.25 µg/mL for benzalkonium chloride, and 1.2 µg/mL for tributyltin chloride. The tested concentration ranges were based on preliminary range-finding experiments and previous ecotoxicological studies (15,16,17) in accordance with standard testing guidelines (18). After the incubation, the viability of nauplii was determined with a magnifying glass based on their motility at 4, 24, and 48 h of exposure. Experiments included a negative control, solvent control (DMSO of 0.1 %), and positive control (potassium dichromate, whose highest tested concentration was 60 µg/mL). Each treatment was done in triplicate. Based on the viability of specimens at certain concentrations, a linear curve was constructed from which the median lethal concentration (LC_50_) was determined by fitting a dose-response curve to mortality data across tested concentrations and by interpolating the concentration corresponding to 50 % lethality, following methods described in standardised ecotoxicological protocols (19, 20). The reliability of the assay was confirmed by the low mortality rate (<10 %) in the negative control group.

### Comet assay

**Figure 2:**
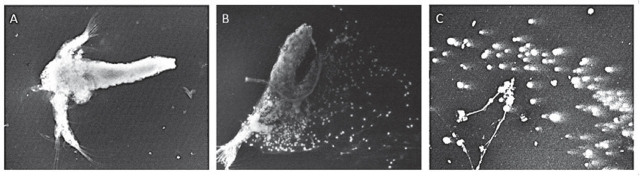
Fluorescence microscopy of A) *A. salina* nauplii embedded in agarose (100× magnification); B) nauplii partially broken into cells (100× magnification), and C) nauplii completely broken in cell suspension (200× magnification)

Each 50 mL polystyrene beaker received 50 nauplii in 20 mL of seawater with selected non-toxic concentrations of cisplatin (6.3, 12.5, and 25 µg/mL), etoposide (9.2, 18.4, and 36.8 µg/mL), and 5-fluorouracil (250, 500, and 1000 µg/mL). Treatments were performed in triplicate in the dark at 25 °C for 24 h. Slides for the comet assay were prepared by adapting the protocol of Eleršek et al. (21) for zebrafish embryos. On slides pre-coated with 1 % NMP, an additional supportive layer was formed by spreading 80 µL of NMP using a coverslip. Nauplii were harvested with phototaxis and transferred to a 2 mL microtube. Seawater was carefully pipetted out to leave nauplii in the residual 100 µL. To each microtube, 100 µL of 1.5 % LMP was added and mixed with a pipette tip. From each tube, 70 µL of the mixture was placed on slides to obtain 5–10 nauplii per gel. Gels were covered with a coverslip and gently pressed to squash nauplii in agarose (Figure 2).

The slides were left to solidify at 4 °C. Coverslips were removed and the slides immersed in cold lysis solution (2.5 mol/L NaCl, 100 mmol/L EDTA, 10 mmol/L Tris, 1.5 % Triton X-100, and 10 % DMSO, pH 10) at 4 °C for 1 h. DNA was denatured in freshly prepared alkaline buffer (300 mmol/L NaOH, 1 mmol/L EDTA, pH 13) at 4 °C for 20 min, followed by electrophoresis under the same conditions (0.5 V/cm, 300 mA, 20 min). Subsequently, the slides were neutralised in 0.4 mol/L Tris buffer (pH 7.5) for 15 min. Before analysis, nucleoids were stained with acridine orange (20 µL, 2 µg/mL per slide) according to Kračun-Kolarević et al. (8). The slides were examined using a Leica DMLS fluorescence microscope (Leica Mikrosysteme GmbH, Vienna, Austria) at 400x magnification, excitation filter 510–560 nm, barrier filter 590 nm, and DNA damage quantified using the Comet Assay IV software (Perceptive Instruments, Stone, UK). For each treatment, two gels were prepared and a total of 150 nucleoids analysed per experimental group. Tail intensity (TI %) was chosen as a descriptor of DNA damage.

### Ethics statement

This study did not involve vertebrates or regulated animal species; therefore, ethical committee approval was not required. *A. salina* nauplii used in toxicity and genotoxicity assays are exempt from the Directive 2010/63/EU and national animal welfare regulations (22). All procedures were performed following institutional guidelines for safe handling of aquatic invertebrates.

### Statistical analysis

Statistical analysis was run on Statistica 7.0 (StatSoft, Inc., Tulsa, OK, USA). For LC_50_ data, we used the regression analysis, and for the comet assay data Kolmogorov-Smirnov test to determine the normality of distribution. A pairwise comparison using Kruskal-Wallis, followed by the Mann-Whitney *U* test, was used to determine the differences between tail intensities between cytostatic-treated groups and corresponding controls. The significance level for all comparisons was set at P<0.05.

To compare the acute toxicity of selected environmental pollutants and the acute toxicity and genotoxicity of the tested chemotherapeutics, raw experimental data were normalised and visualised with a heatmap in the R version 4.3.2 (Institute for Statistics and Mathematics, Vienna, Austria) using the pheatmap and dplyr packages. Since lower LC_50_ values or genotoxic concentration (the lowest concentration with TI % significantly higher than control) indicate higher toxicity/genotoxicity, an inverse transformation was applied to obtain a directly proportional toxicity index (1/LC_50_) or genotoxicity index (1/genotoxic concentration).

To enable comparison between variables expressed on different scales, both the toxicity and genotoxicity indices were normalised using min-max scaling according to the following equation:

[1] $${\text{X}}_{\text{normalised}}=[\text{X}-\min(\text{X})]/[\max(\text{X})-\min(\text{X})]$$

## RESULTS AND DISCUSSION

### Acute toxicity findings

Table 1 shows the mean acute toxicities of glyphosate, benzalkonium chloride, and tributyltin chloride in *A. salina* over 4, 24, and 48 h time-points. Glyphosate produced moderate acute toxicity toward *A. salina* nauplii, with LC_50_ values comparable to those reported previously. However, De Brito Rodrigues et al. (17) observed lower LC_50_ values for glyphosate formulations on *A. salina* (the 48-hour LC_50_ values were 14.19 µg/mL for the Roundup formulation and 37.53 µg/mL for glyphosate AKB 480), indicating that both formulations were significantly more toxic than the isolated active ingredient, primarily due to the presence of additional compounds, particularly the surfactant tallow amine.

**Table 1:** Mean acute toxicity of tested environmental pollutants on *A. salina*

**LC_50_**	**Glyphosate (µg/mL)**	**Benzalkonium chloride (µg/mL)**	**Tributyltin chloride (µg/mL)**	Potassium dichromate (K_2_Cr_2_O_7_)* (µg/mL)
4 h	160.58±7.77	14.15±3.60	>1.2	40.60±1.78
24 h	128.20±17.78	12.38±1.19	0.63±0.03	38.50±3.69
48 h	42.79±12.44	7.45±2.07	0.55±0.01	21.54±3.86

* – positive control

Benzalkonium chloride, a commonly used quaternary surfactant, exhibited a substantially higher toxic effect compared to glyphosate, indicating that it poses a considerable risk to aquatic microfauna. In their study on *Artemia franciscana* larvae, Bartolomé and Sanchez-Fortún (23) determined the LC_50_ values of 32.67 µg/L, 9.14 µg/L, and 0.7 ng/L for 24, 48, and 72-hour incubation periods, respectively. The discrepancy between our study and their observations can be attributed to differences in developmental stage, exposure duration, and experimental conditions. In addition, factors such as salinity and test medium composition are known to influence surfactant toxicity, which may further explain the higher LC_50_ values observed in our study.

However, *A. salina* may be less sensitive to benzalkonium chloride compared to other standard test organisms, which should be considered when interpreting toxicity data. For instance, *D. magna* and *Ceriodaphnia dubia* have demonstrated significantly higher sensitivity to benzalkonium chloride (LC_50_ of 32.2 µg/L for *D. magna* and 403.7 µg/L for *C. dubia*) (24). These observations highlight the importance of species selection in ecotoxicological assessments, as sensitivity to toxicants can vary substantially among aquatic organisms.

Tributyltin chloride, a lipophilic and bioaccumulative compound, resulted as the most toxic, which is consistent with the well-documented persistence, lipophilicity, and bioaccumulative nature of organotin compounds in aquatic environments (25, 26) and with its ability to disrupt cellular membranes, impair mitochondrial function, and induce oxidative stress (27). In *D. magna* as a model organism (15), no significant mortality was observed until prolonged exposure, with a 96 h LC_50_ of 5.9 ng/mL. Lower tributyltin chloride concentrations resulted in sublethal effects such as changes in swimming activity and behaviour (15, 28). The higher LC_50_ values we obtained can be attributed to differences in exposure duration, assessed endpoints (i.e. lethal effects in this study vs behavioural and sublethal changes in previous studies), and species-specific sensitivity. Additionally, *D. magna* is generally more sensitive than *A. salina* (29).

Potassium dichromate (K_2_Cr_2_O_7_), used as a positive control, showed LC_50_ values consistent with previously reported data (30, 31), confirming the reliability and sensitivity of the applied bioassay.

Interestingly, reports from the literature suggest much higher sensitivity of *Daphnia magna* to potassium dichromate (32) as well as benzalkonium chloride (24) and tributyltin chloride (15, 28, 29) than *A. salina* and further highlight the importance of using multiple test organisms in ecotoxicological risk assessment to capture a broader spectrum of potential environmental effects.

**Figure 3:**
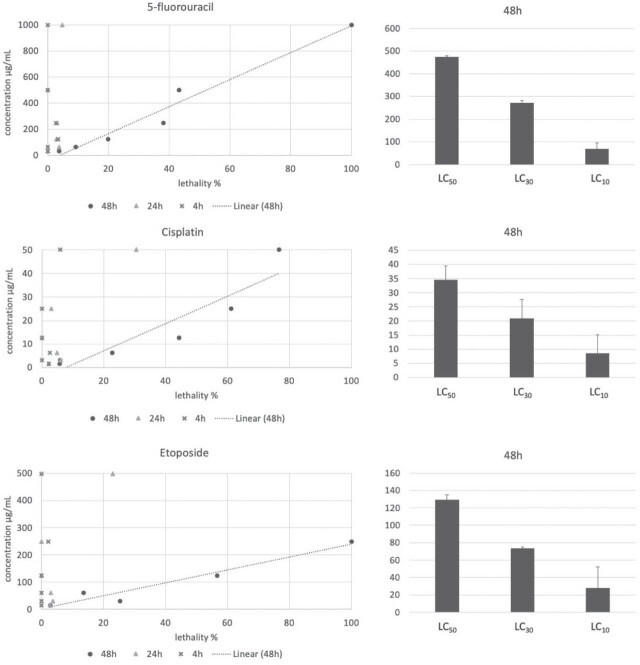
5-fluorouracil, cisplatin, and etoposide toxicity in *A. salina* over 4 h, 24 h, and 48 h of incubation. Lines represent a trend according to the toxicity results obtained for each concentration for a 48 h incubation period that was used to determine mean (±SD) LC_50_, LC_30_, and LC_10_ values (right)

Figure 3 shows the concentration-dependent effects of the three cytostatics on *A. salina* mortality over 4 h, 24 h, and 48 h, but median lethality was achieved only after 48 h of exposure. These endpoints provide insight into lethal, sublethal, and low-effect toxicity, enabling a comparative assessment of compound potency, while LC_10_ and LC_30_ further support evaluation of environmentally relevant and mechanistic (e.g. genotoxic) effects, enabling a more detailed insight into toxic responses relevant for ecotoxicological risk assessment.

With the 48-hour LC_50_ of 474.57 µg/mL, 5-fluorouracil exhibited the lowest, and cisplatin the highest toxicity (LC_50_ 34.61 µg/mL). These findings are consistent with the well-documented mechanism of cisplatin, which involves DNA cross-linking, leading to disruption of cellular replication and induction of apoptosis (33). Etoposide toxicity was in between (LC_50_=129.11 µg/mL), which aligns with its mechanism of action inhibiting topoisomerase II and resulting in DNA strand breaks with a somewhat delayed onset compared to direct DNA alkylating agents (34).

Comparison with previous studies further highlights interspecies variability. Parrella et al. (16) reported substantially lower LC_50_ values for a 48 h incubation period for *D. magna* (e.g. 0.94 µg/mL for cisplatin and 20.84 µg/mL for 5-fluorouracil), indicating higher sensitivity compared to *A. salina*. Similarly, *C. dubia* (24 h incubation period) showed greater sensitivity to cisplatin with 13.5 times lower LC_50_ value (2.5 µg/mL), while responses to 5-fluorouracil were comparable (16). These findings highlight the importance of using multiple model organisms in ecotoxicological assessments. However, no LC_50_ could be determined for etoposide in *D. magna*, likely due to solubility limitations that prevented testing at sufficiently high concentrations, while in *C. dubia*, only a 16 % mortality was observed at the highest tested concentration (120 mg/L) (16). More importantly, although cytostatics are designed to exert biological effects at low concentrations, the LC_50_ values obtained in this study are several orders of magnitude higher than those typically detected in aquatic environments (ng/L to low µg/L range) and primarily reflect intrinsic toxic potential under controlled conditions rather than direct environmental risk.

### Genotoxicity findings

**Figure 4:**
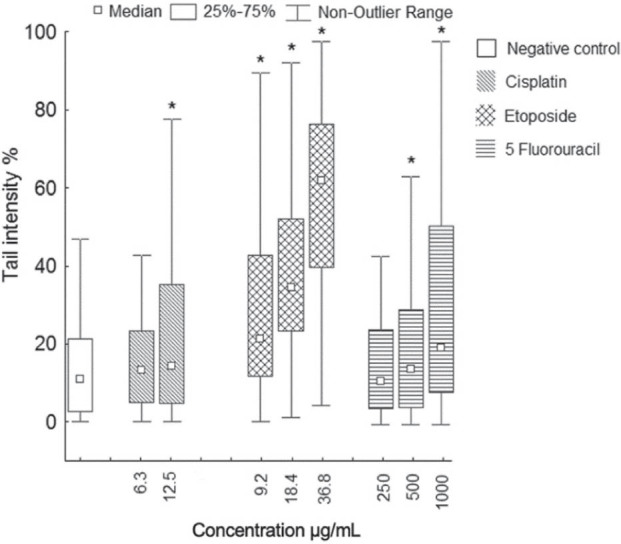
Level of DNA damage measured by tail intensity (%) in the cells of *A. salina* (150 analysed nucleoids per group). *significant increase in DNA damage compared to negative control (P<0.05; Kruskal-Wallis and Mann-Whitney *U* test)

Figure 4 shows tail intensities (TI %) as the measure of genotoxic effects of the three cytostatics in *A. salina*. All induced a clear concentration-dependent increase in DNA damage in the following order: etoposide > 5-fluorouracil > cisplatin. Compared to the unexposed control, cisplatin induced a significant increase in tail intensity only at 12.5 µg/mL, but this result should be interpreted with caution, as cisplatin forms DNA cross-links that can reduce DNA migration during electrophoresis and yield an underestimation of DNA damage in the comet assay (35).

A similar concentration-dependent increase in DNA damage was observed with 5-fluorouracil, reaching significance at much higher concentrations (500 and 1000 µg/mL). This response is consistent with its role as a pyrimidine analogue, which interferes with DNA synthesis and repair, leading to indirect genotoxic effects that may require higher exposure levels to become pronounced (36).

In contrast, etoposide exhibited the strongest genotoxic effect, with tail intensity significantly higher than control at all concentrations. This is consistent with its mechanism of action as a topoisomerase II inhibitor, which induces DNA strand breaks and leads to the accumulation of DNA damage. Notably, similar DNA damage of approximately 40 % tail intensity has been reported at 19.42 µg/mL in MRC-5 cells (37), supporting the relevance of the findings obtained in *A. salina*.

We believe that the observed differences in genotoxic effects between the three cytostatics reflect their distinct mechanisms of action. Cisplatin forms DNA cross-links (38), etoposide inhibits topoisomerase II, leading to DNA strand breaks (39), and 5-fluorouracil acts as a pyrimidine analogue, interfering with DNA synthesis (36).

Several studies have demonstrated that *A. salina* can serve as a reliable model organism for preliminary toxicity and genotoxicity screening. For example, Rajabi et al. (40) reported no significant differences between *A. salina* lethality tests and MTT assays on L929 mouse fibroblast cells, while Albarano et al. (41) highlighted that DNA damage responses in the *Artemia* species are comparable to those observed in other biological models, including mammalian systems.

Compared to other models, *A. salina* exhibited lower sensitivity to cisplatin and 5-fluorouracil, while the genotoxic effect of etoposide was comparable.

### Combined cytostatic toxic and genotoxic effects

**Figure 5:**
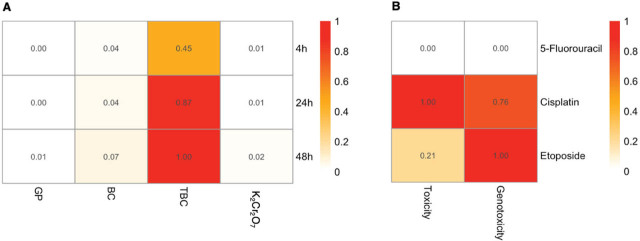
Normalised heatmap comparing A) the acute toxicity (expressed as inverse LC_50_ values) of glyphosate (GP), benzalkonium chloride (BC), and tributyltin chloride (TBC) and B) the acute toxicity and genotoxicity of three 5-fluorouracil, cisplatin, and etoposide. Higher values (red) represent greater biological effect

Interestingly, despite its apparently lower genotoxicity in the comet assay, cisplatin showed the strongest combined toxic and genotoxic effect, as illustrated by the integrated heatmap (Figure 5B). This can be attributed to its multiple modes of action, including DNA cross-linking, disruption of replication, oxidative stress, and mitochondrial dysfunction, ultimately leading to apoptosis (33).

Among all tested environmental pollutants, tributyltin chloride exhibited the highest toxicity toward *A. salina* across all incubation periods.

### The hazard potential of the obtained toxicity values

All findings should be interpreted in the context of environmentally relevant concentrations. The LC_50_ values obtained in this study (tens to hundreds of mg/L) are several orders of magnitude higher than concentrations typically reported in aquatic environments. Environmental concentrations of cytostatics such as 5-fluorouracil and etoposide are generally in the ng/L range (42, 43), while cisplatin has been detected in hospital effluents at up to low µg/L levels (44, 45). Similarly, tributyltin chloride is currently found at ng/L concentrations due to regulatory restrictions (45), and benzalkonium chloride typically occurs at low µg/L levels in surface waters (46). Glyphosate, although more prevalent, is most commonly detected in surface waters at µg/L concentrations (47). Thus, the concentrations required to induce acute toxicity in *A. salina* in this study exceed environmentally relevant levels by approximately 10^3^ to 10^6^ times, depending on the compound.

Nevertheless, the obtained results remain relevant from an ecotoxicology point of view, as they provide comparative insights into the intrinsic toxicity and relative potency of the tested compounds, which is essential for hazard ranking. Moreover, compounds such as cytostatics can exert biological effects at concentrations well below those causing acute mortality, particularly due to their specific modes of action, including DNA interaction and disruption of cellular processes. Therefore, the integration of acute toxicity data with sublethal endpoints, such as genotoxicity, is crucial for a more comprehensive assessment of their ecological risk.

## CONCLUSIONS

Even though *A. salina* seems to be less sensitive than freshwater models such as *D. magna*, it showed a reliable response in both acute toxicity and genotoxicity, supporting its suitability as a fit-for-purpose model for preliminary hazard screening. It exhibited high sensitivity to etoposide, comparable to human cell lines, while the lower responses to cisplatin and 5-fluorouracil indicate compound-specific variability. In acute toxicity tests, relatively high LC_50_ values suggest lower sensitivity compared to some standard aquatic organisms, highlighting interspecies differences. Therefore, *A. salina* should be regarded not as a universally sensitive model but as a practical and efficient screening tool, whose performance depends on the mode of action of the tested compound.

To address the limited scope of our study, which relied on one *Artemia* species, only assessed the genotoxicity of cytostatics, and focused exclusively on acute exposure scenarios, future studies should include additional test species, broader genotoxic screening, and chronic or mixture exposures to improve ecological relevance.
